# Pulmonary Complications of Rheumatoid Arthritis Presenting as Spontaneous Pneumothorax and Empyema: A Case Report

**DOI:** 10.7759/cureus.101407

**Published:** 2026-01-12

**Authors:** Roshni Jagaram, Subramanian Nallasivan

**Affiliations:** 1 Medicine, Velammal Medical College Hospital and Research Institute, Madurai, IND; 2 Rheumatology, Velammal Medical College Hospital and Research Institute, Madurai, IND

**Keywords:** case report, empyema, immunosuppressive therapy, interstitial lung disease, rheumatoid arthritis, spontaneous pneumothorax

## Abstract

Rheumatoid arthritis (RA) is a systemic autoimmune disease with well-recognized pulmonary manifestations. While interstitial lung disease and pleural effusions are common, spontaneous pneumothorax complicated by empyema is infrequently reported and may pose significant diagnostic and therapeutic challenges, particularly in immunocompromised patients. We report the case of a 58-year-old woman with long-standing RA and diabetes mellitus who presented with acute dyspnea and pleuritic chest pain. Imaging revealed a right-sided spontaneous pneumothorax with pleural effusion. Despite intercostal drainage and broad-spectrum intravenous antibiotics, the patient developed a loculated empyema requiring surgical decortication. This case highlights the importance of early imaging, close monitoring of chest tube response, and timely surgical referral in complex pleural infections associated with RA.

## Introduction

Rheumatoid arthritis (RA) is a chronic autoimmune disease primarily affecting synovial joints but frequently associated with extra-articular involvement, particularly of the lungs. Pulmonary manifestations occur in up to 60% of patients during the disease course and include interstitial lung disease, pleural effusions, airway disease, pulmonary nodules and less commonly, spontaneous pneumothorax [1,2].

Spontaneous pneumothorax in RA has been attributed to rupture of subpleural nodules, necrobiotic lung lesions, or fragile lung parenchyma, especially in patients receiving long-term immunosuppressive therapy [3]. Superimposed infection of pleural collections may lead to empyema, a condition associated with increased morbidity and mortality, particularly in patients with diabetes mellitus or impaired immunity [4]. The progression from pneumothorax to loculated empyema in RA remains poorly reported, with most available evidence limited to isolated case reports. We present a case that illustrates this uncommon but clinically significant complication.

## Case presentation

A 58-year-old woman with a 10-year history of seropositive RA presented with acute-onset dyspnea (New York Heart Association class II) and right-sided pleuritic chest pain for two days. Her medical history was significant for type 2 diabetes mellitus and vasculitic neuropathy. She had been receiving long-term disease-modifying antirheumatic therapy along with intermittent corticosteroids.

On admission, she was tachypneic with a respiratory rate of 28 breaths/min, oxygen saturation of 89% on room air, heart rate of 104 beats/min, and blood pressure of 130/80 mmHg. Chest examination revealed reduced breath sounds over the right hemithorax with basal crepitations.

Laboratory evaluation (Table 1) showed anemia (hemoglobin 9.5 g/dL) and marked leukocytosis (38,200 cells/cu.mm). Inflammatory markers were elevated, including procalcitonin (6.59 ng/mL) and D-dimer (506 ng/dL). Renal and hepatic function tests were within normal limits. Glycemic control was suboptimal (HbA1c 7.9%). Rheumatoid factor was elevated at 160 IU/L, and the anti-cyclic citrullinated peptide antibody level was 120.8 U/mL. Antinuclear antibody testing was negative. Cardiac workup (ECG, troponin I, CK-MB) was within normal limits.

**Table 1:** Laboratory Investigations Anti-CCP: Anti-cyclic citrullinated peptide; AFB: acid-fast bacillus; LDH: lactate dehydrogenase; ADA: adenosine deaminase; Pro-BNP: B-type natriuretic peptide; ALT: alanine transaminase; AST: aspartate aminotransferase; ALP: alkaline phosphatase

Investigations	Reports	Reference Range
CBC		
Hemoglobin	9.5 g/dl	13 – 17g/dl
Total leukocyte count	38,200 cells/cu.mm	4,000 – 11,000 cells/cu.mm
Platelets	4,19,000/cu.mm	1,50,000-4,50,000/cu.mm
HbA1c	7.9 %	<5.7%
Renal Function Test		
Blood urea	71 mg/dl	18-45 mg/dl
Creatinine	1.2 mg/dl	0.5 – 0.9 mg/dl
Liver Function Test		
AST	10U/L	0-35 U/L
ALT	16U/L	0-35 U/L
ALP	130U/L	30 – 120 U/L
Protein	6.7 g/dl	6.0-8.3 g/dl
Procalcitonin	6.59 ng/ml	0 – 0.1ng/ml
NT Pro-BNP	443.7 pg/ml	0 – 125pg/ml
D-Dimer	506ng/dl	<500ng/dl
Pleural Fluid		
ADA	156.1 U/L	<40 U/L
PH	7.0	>7.60-7.64
LDH	21310 IU/L (1:10 dilution)	<200 IU/L
Glucose	<40mg/dl	>60mg/dl
Protein	5.1 g/dl	<3.0 g/dl
GeneXpert	MTB not detected	Negative
AFB stain	No acid-fast bacilli	Negative
Pleural fluid cytology	Dense neutrophil-rich smear	-
Pleural fluid total count	64,000 cells/cu.mm	<1000 cells/cu.mm
Pleural fluid culture	No growth	Sterile/ no growth
Blood culture	No growth	Sterile/ no growth
Rheumatoid factor	160IU/L	<10IU/L
Anti-CCP	120.8 U/ml	<20 U/ml
Anti-nuclear antibody	Negative	Negative
ECG	Sinus rhythm	
Trop I	Negative	Negative
CK-MB	<3ng/ml	<5ng/ml

**Figure 1:**
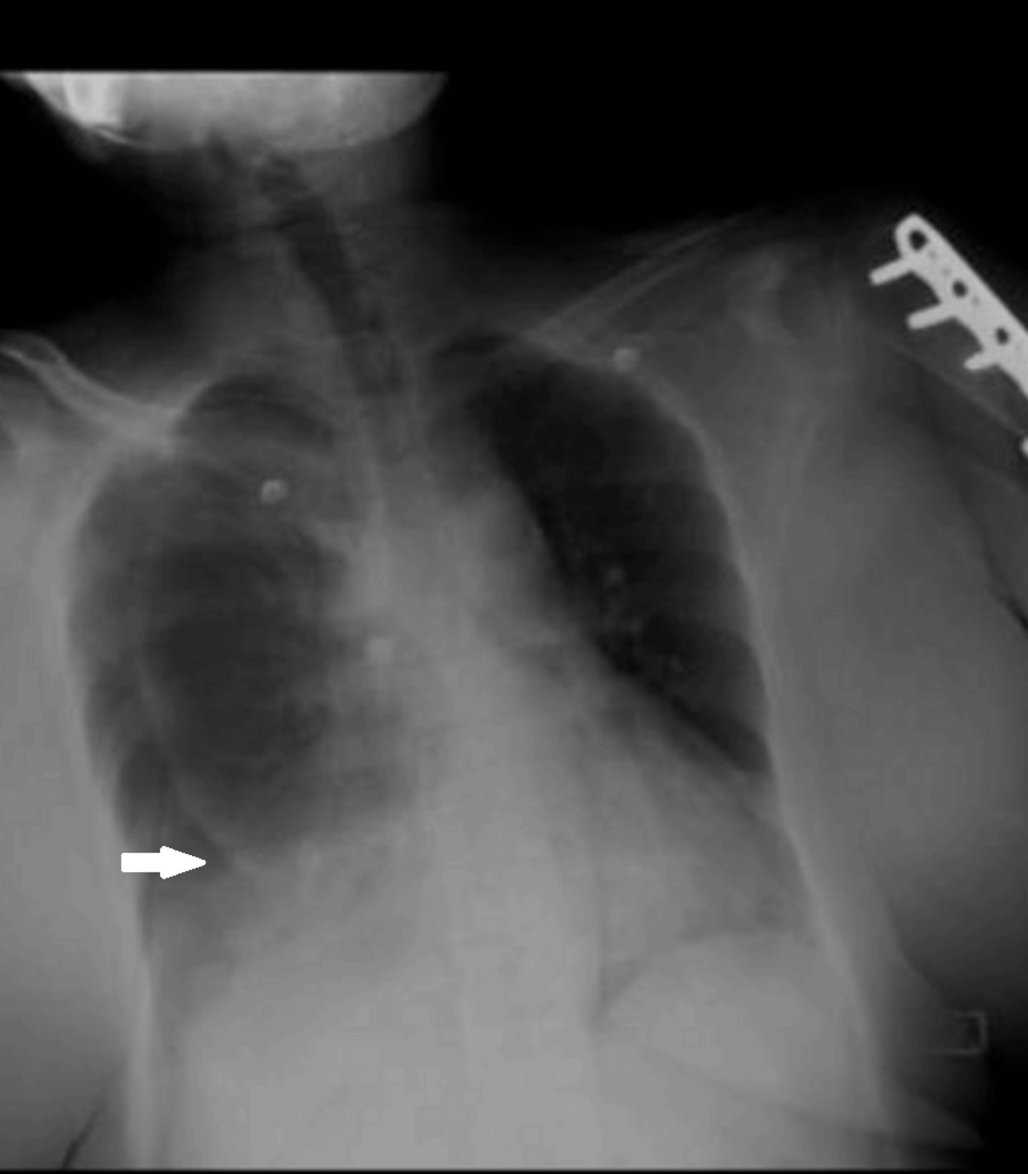
Chest radiograph (PA view) demonstrating a right-sided pneumothorax with partial lung collapse and reduced lung markings peripherally.

**Figure 2:**
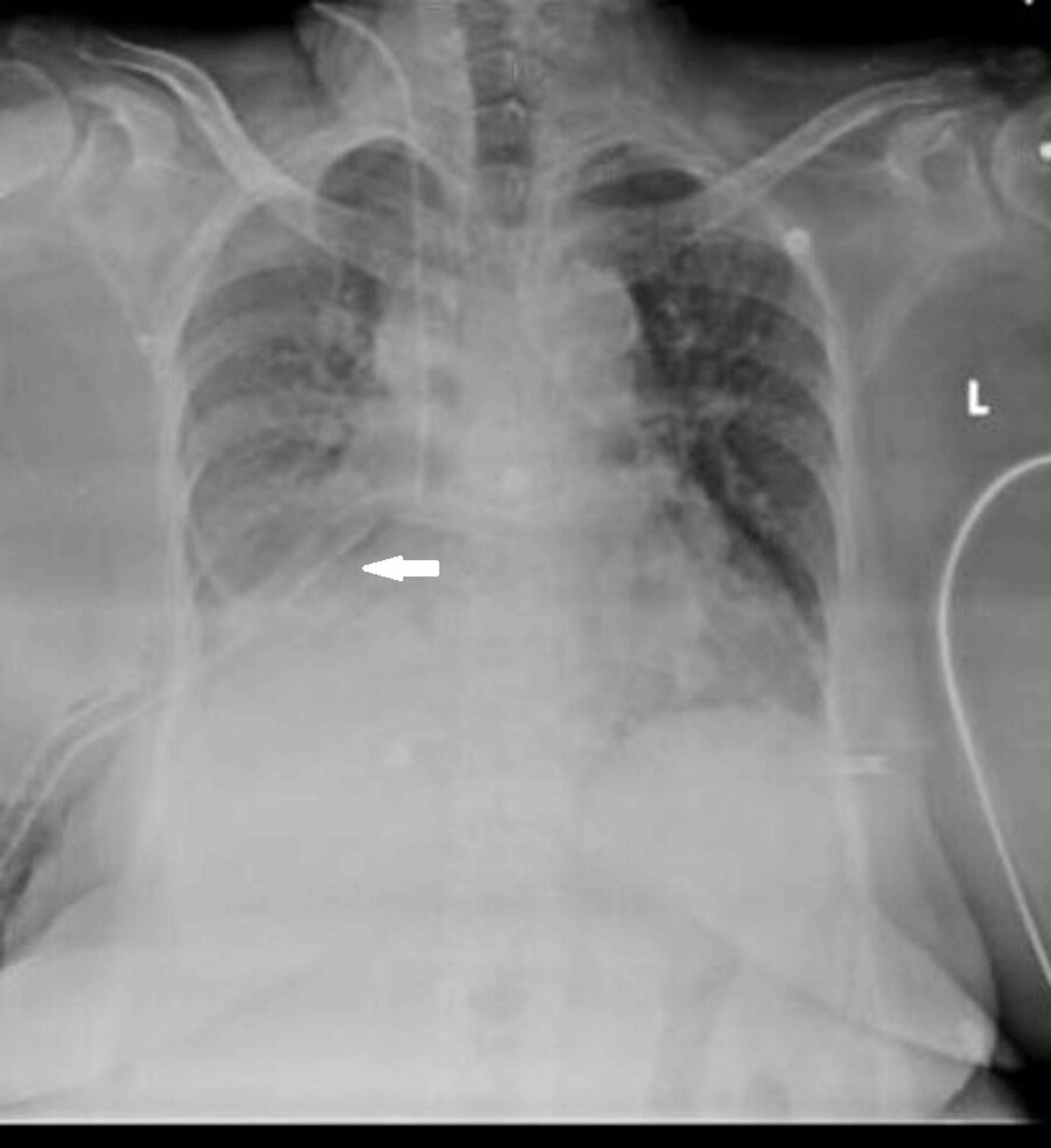
Chest radiograph (PA view) following intercostal drain insertion, showing persistent right-sided pneumothorax with incomplete lung expansion.

A chest X-ray obtained on the day of admission demonstrated a moderate right-sided pneumothorax (Figure 1). An intercostal drain (ICD) was inserted immediately (Figure 2), but clinical improvement was minimal, characterized by persistent tachypnea, ongoing oxygen requirement, continued chest discomfort, and lack of significant radiographic lung re-expansion.

**Figure 3:**
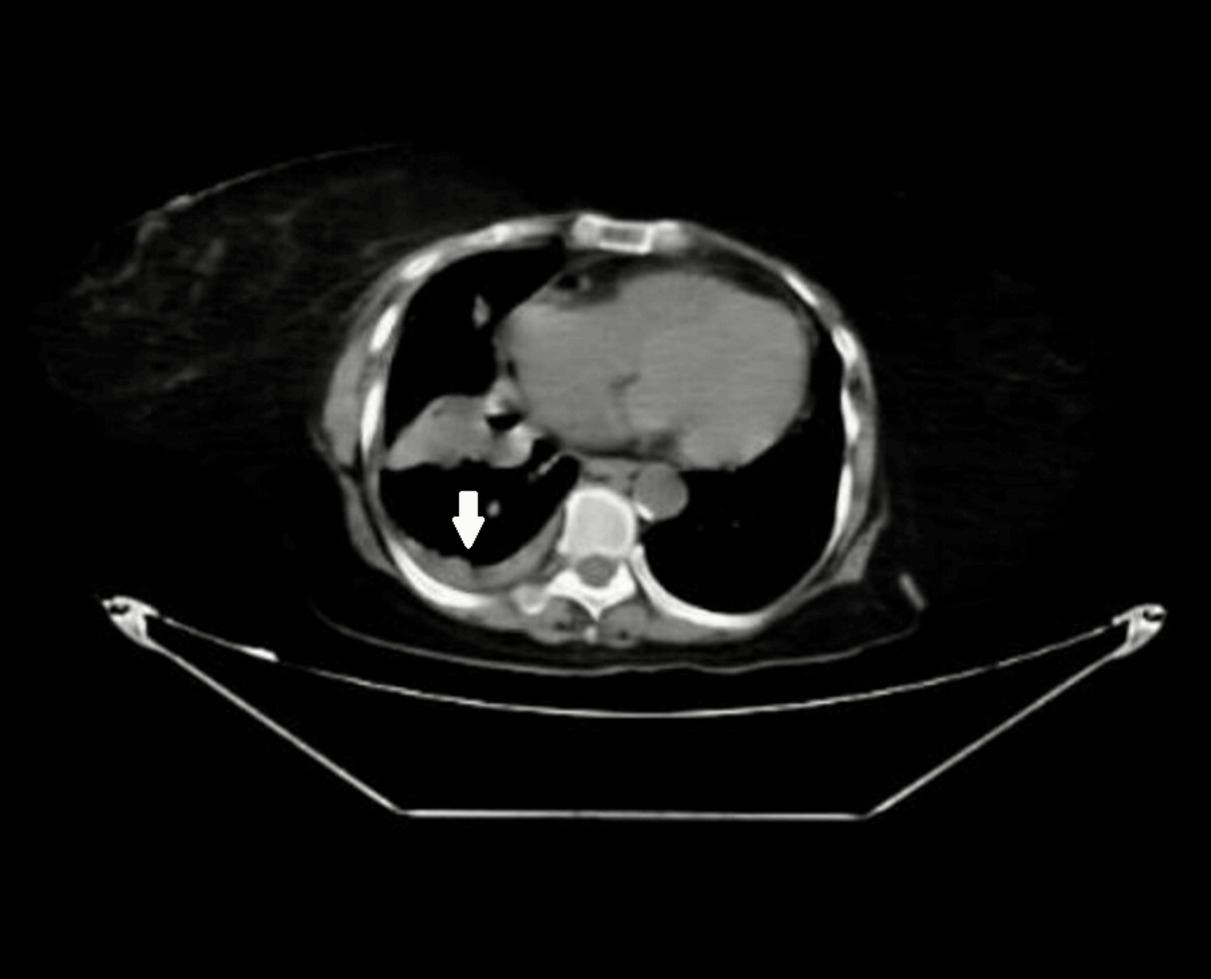
Contrast-enhanced CT chest demonstrating right-sided pleural thickening with moderate pleural effusion and persistent pneumothorax, suggestive of impaired pleural drainage.

Contrast-enhanced computed tomography of the chest performed two days after ICD insertion revealed a persistent pneumothorax with pleural thickening and moderate pleural effusion, suggestive of impaired drainage (Figure 3).

Pleural fluid analysis obtained via the ICD was exudative as per Light’s criteria, with low glucose (<40 mg/dL; reference range: >60 mg/dL), markedly elevated lactate dehydrogenase (21,310 IU/L after dilution; reference range: <200 IU/L and/or < two-thirds of the upper limit of normal serum lactate dehydrogenase), neutrophilic predominance, and acidic pH (7.0), consistent with empyema. Microbiological cultures, acid-fast bacilli staining, and GeneXpert testing were negative.

The patient was initiated on broad-spectrum intravenous antibiotics to cover gram-positive, gram-negative, and anaerobic organisms. Empiric therapy included intravenous piperacillin-tazobactam, with additional gram-positive coverage given the patient’s immunocompromised state and severity of illness. Antibiotic therapy was continued for approximately two weeks and monitored using clinical parameters and inflammatory markers.

**Figure 4:**
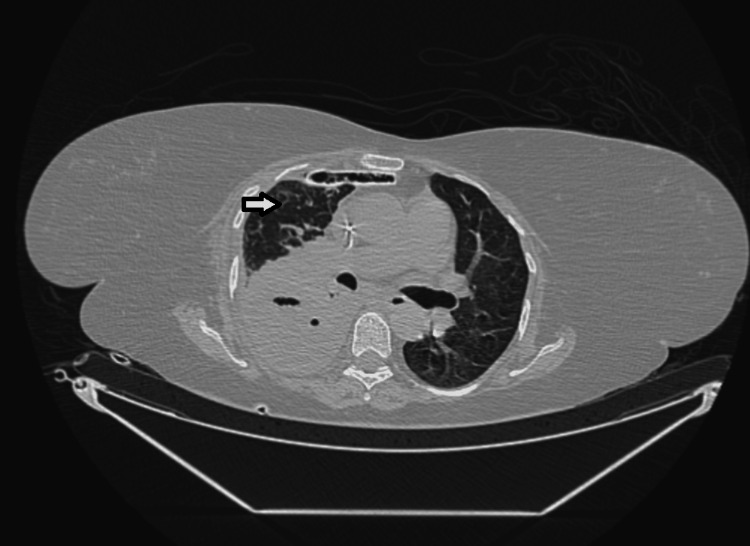
Contrast-enhanced CT chest showing loculated pleural collection with air–fluid levels and pleural thickening, consistent with loculated empyema.

Despite appropriate antimicrobial therapy and intercostal drainage, the patient developed a loculated empyema, confirmed on repeat CT imaging (Figure 4). The failure of ICD drainage was attributed to pleural thickening and early loculation, preventing adequate evacuation of infected fluid.

Given persistent symptoms and radiological progression, the patient underwent right posterolateral mini-thoracotomy with lung decortication on hospital day seven. Intraoperatively, dense fibrous adhesions and loculated purulent collections were identified. Complete decortication allowed full lung re-expansion.

**Figure 5:**
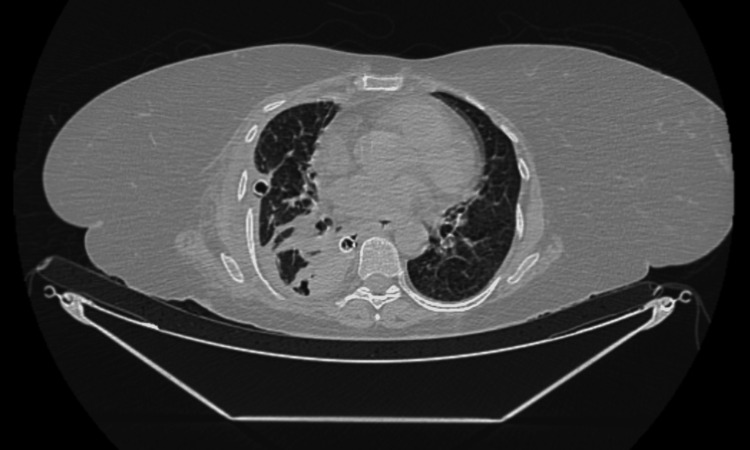
Follow-up CT chest demonstrating complete lung re-expansion with resolution of pneumothorax and pleural collections following surgical decortication.

Postoperatively, the patient demonstrated steady clinical improvement, with resolution of oxygen requirements and declining inflammatory markers. Corticosteroid therapy was adjusted perioperatively, and glycemic control was optimized. Serial imaging showed resolution of the pneumothorax and empyema (Figure 5).

At the time of discharge, the patient was clinically stable, afebrile, maintaining oxygen saturation on room air, with normal blood pressure and heart rate and no respiratory distress. She remained asymptomatic on follow-up.

## Discussion

Pulmonary involvement in RA contributes significantly to morbidity and mortality. While spontaneous pneumothorax is uncommon, it has been described in association with subpleural rheumatoid nodules and necrobiotic lung lesions [3,5]. Immunosuppression and corticosteroid therapy may further predispose patients to secondary infections and delayed tissue repair.

Empyema in RA patients poses additional challenges, particularly in the presence of diabetes mellitus, which is associated with impaired neutrophil function and increased susceptibility to severe infections [4,6]. In this case, early loculation and pleural thickening likely contributed to ICD failure, emphasizing that persistent symptoms or poor radiographic response after chest tube placement should prompt early cross-sectional imaging.

Surgical decortication remains the definitive treatment for loculated empyema when conservative management fails and has been associated with favorable outcomes when performed in a timely manner [7]. This case underscores the importance of a multidisciplinary approach and early surgical referral in complex pleural infections in immunocompromised patients.

## Conclusions

Spontaneous pneumothorax complicated by empyema represents a serious pulmonary manifestation of RA. Clinicians should maintain a high index of suspicion in RA patients presenting with acute respiratory symptoms, particularly in the setting of immunosuppression and metabolic comorbidities. Early imaging, prompt initiation of empiric broad-spectrum antibiotics, careful monitoring of chest tube response, failure of lung re-expansion, persistent systemic inflammation or radiological evidence of loculated collections within the first few days should prompt early multidisciplinary discussion (involving rheumatologists, pulmonologists, physicians and cardiothoracic surgeons) and timely surgical referral. Optimization of underlying RA disease activity is crucial to reduce the risk of recurrent or progressive pulmonary complications and improve long-term outcomes. Clinicians should be aware of these uncommon manifestations to facilitate prompt diagnosis and reduce morbidity and mortality.
